# Branchioma with a nested/organoid morphology: molecular profiling of a distinctive potentially misleading variant and reappraisal of potential relationship to CD34-positive/Rb1-deficient tumors of the neck

**DOI:** 10.1007/s00428-023-03592-9

**Published:** 2023-07-04

**Authors:** Martina Baněčková, Michael Michal, Tomáš Vaněček, Petr Grossman, Dimitar Hadži Nikolov, Radek Včelák, Radim Žalud, Michal Michal, Abbas Agaimy

**Affiliations:** 1 Sikl’s Department of Pathology, Faculty of Medicine, Charles University, E. Benese 13, 305 99 Pilsen, Czech Republic; 2 Bioptic Laboratory, Ltd, Pilsen, Czech Republic; 3 Molecular and Genetic Laboratory, BiOptic Laboratory, Ltd., Pilsen, Czech Republic; 4 Pathology Department, Regional Hospital Kolin JSC, Kolin, Czech Republic; 5 Institute of Pathology, University Hospital Erlangen, Friedrich-Alexander University Erlangen-Nürnberg (FAU), Erlangen, Germany

**Keywords:** Branchioma, Ectopic hamartomatous thymoma, Neuroendocrine carcinoma-like, Head and neck, Retinoblastoma, *RB1* gene, CD34, Androgen receptor

## Abstract

Branchioma (previously called ectopic hamartomatous thymoma, branchial anlage mixed tumor, or thymic anlage tumor) is a rare lower neck lesion with an adult male predominance and an uncertain histogenesis. Except for 4 cases, all branchiomas described in the literature were benign. Recently, *HRAS* mutation was detected in one case, but still little is known about the molecular genetic background of this rare entity. We herein report the histological, immunohistochemical, and molecular genetic analysis of a branchioma with a nested/organoid (neuroendocrine-like) morphology in a 78-year-old man. Histology revealed classical branchioma areas merging with nested/organoid cellular component lacking conventional features of malignancy. Immunohistochemistry was positive for high-molecular-weight cytokeratins. CD34 was expressed in the spindle cell component. Moreover, the tumor cells showed near-complete loss of retinoblastoma (RB1) expression (<1% of cells positive). All neuroendocrine markers (synaptophysin, chromogranin, and INSM1) were negative. Next-generation sequencing (TSO500 Panel) revealed 5 pathogenic/likely pathogenic mutations including 1 mutation in *KRAS* and 2 different mutations in each of *MSH6* and *PTEN.* FISH and DNA sequencing were negative for *RB1* gene alterations. To our knowledge, this is the first report of a branchioma showing misleading nested/organoid morphology and the first report on Rb1 immunodeficiency in this entity, in addition to multiple gene mutations revealed by NGS.

**Supplementary Information:**

The online version contains supplementary material available at 10.1007/s00428-023-03592-9.

## Introduction

Branchioma is a rare site-specific benign tumor with an adult male predominance, typically occurring in the lower neck and combining bland squamoid epithelial with variable mesenchymal elements, reminiscent of thymic tissue, hence the widely used historical terminology “ectopic hamartomatous thymoma” [1]. Since its first description by Smith and McClure in 1982 [2] and later by Rosai et al. [3], branchioma was reported in many case series and single case reports under different names such as ectopic hamartomatous thymoma, branchial anlage mixed tumor or thymic anlage tumor, and biphenotypic branchioma [4–6]. In the upcoming WHO classification of the head and neck tumors that is currently in beta version, the name “branchioma” is adopted for this neoplasm [7].

Branchioma is composed of endodermal and mesodermal lineage derivatives, particularly, of epithelial islands, spindle cells, and mature adipose tissue [7], but no tissue of genuine thymic origin or thymic differentiation was described so far [4–6]. Epithelial cells are arranged in non-keratinizing epithelial islands, cysts, or glandular structures, sometimes with a peripheral rim of residual myoepithelial cells. The spindle cells are plump and arranged in haphazard, storiform, or fascicular patterns with intermingled thick collagen. Sometimes, the spindle-shaped cells grow in solid sheets with interspersed adipose tissue, imitating a pleomorphic adenoma-like morphology [8]. Both the epithelial and spindled components most likely originate from the same progenitor cell [4] and both show positivity for pan-keratins as well as frequent nuclear androgen receptor (AR) expression, which might explain its prevailing occurrence in postpubertal men [9]. However, the spindle cells also show CD34, p63, and SMA positivity. The latter 2 markers also decorate the myoepithelial cell component, when present [10].

The herein presented case expands the morphological, immunohistochemical, and molecular genetic spectrum of this rare tumor entity.

## Materials and methods

### Histology and immunohistochemistry

The tissue specimen was fixed in formalin, embedded in paraffin, and processed routinely for conventional microscopy using hematoxylin and eosin staining.

For immunohistochemistry, 4-μm-thick sections were cut from paraffin blocks and mounted on positively charged slides (TOMO, Matsunami Glass IND, Osaka, Japan). Sections were processed on a BenchMark ULTRA (Ventana Medical Systems, Tucson, AZ), deparaffinized, and subjected to heat-induced epitope retrieval by immersion in a CC1 solution (pH 8.6) at 95°C. The primary antibodies used in this study are summarized in Table 1. Antigen visualization was performed using the ultraView Universal DAB Detection Kit (Roche, Tucson, AZ) and ultraView Universal Alkaline Phosphatase Red Detection Kit (Roche, Tucson, AZ). The slides were counterstained with Mayer’s hematoxylin. Appropriate positive controls were employed.

**Table 1:** Antibodies used for the immunohistochemical study

Antibody	Clone	Dilution	Antigen retrieval/time	Source
Retinoblastoma 1	G3-245	1:25	CC1/66 min	BD Biosciences
CD34	QBEnd/10	1:200	CC1/ 64 min	Dako Cytomation
AE1–AE3	AE1/AE3	RTU	EnVision High pH/30 min	DAKO
OSCAR	IsoType:IgG2a	1:500	EnVision High pH/30 min	Covance
Smooth muscle actin	1A4	RTU	CC1/36 min	Cell Marque
Ki-67	MIB-1	RTU	EnVision High pH/30 min	DAKO
p63	DAK-p63	RTU	EnVision Low pH/30 min	DAKO
S-100 protein	polyclonal	RTU	EnVision High pH/30 min	DAKO
Androgen receptor	SP107	RTU	CC1/64 min	Cell Marque
INSM1	A-8	1:1000	CC1/ 64 min	Santa Cruz
Synaptophysin	DAK-SYNAP	RTU	EnVision High pH/30 min	DAKO
Chromogranin	DAK-A3	RTU	EnVision Low pH/30 min	DAKO
CD56	123C3	RTU	EnVision High pH/30 min	DAKO
MSH6	SP93	RTU	CC1/64C,	VENTANA

*RTU*, ready to use; *min*, minutes

CC1: EDTA buffer, pH 8.6, 95 °C

EnVision High pH, pH 9.0, 97 °C

EnVision Low pH, pH 6.0, 97 °C

### Molecular genetic study

#### Archer FusionPlex assay

The in-house customized version of Archer FusionPlex Sarcoma kit was used to construct a cDNA library for detecting fusion transcripts and point mutations in 88 and 14 genes (Supplementary File), respectively. The complete list of genes and mutations covered by this assay has been reported previously [11]. All steps were performed according to the manufacturer’s instructions, and the library was sequenced on an Illumina platform as described previously [12].

#### Illumina TruSight Oncology 500 assay

The case was analyzed using the commercially available TruSight Oncology 500 assay from Illumina. This panel analyzes both DNA and RNA. The DNA analysis interrogates 523 genes for single-nucleotide variants (SNVs) and indels and the RNA analysis interrogates 55 genes. The complete list of genes can be found on the manufacturer’s website (https://www.illumina.com/content/dam/illumina-marketing/documents/products/gene_lists/gene_list_trusight_oncology_500.xlsx).

Briefly, DNA libraries were prepared using the TruSight Oncology 500 Kit (Illumina) according to the manufacturer’s protocol, except for DNA enzymatic fragmentation which was done using KAPA Frag Kit (KAPA Biosystems, Washington, MA). Sequencing was performed on the NextSeq 550 sequencer (Illumina) following manufacturer’s recommendations. Data analysis (DNA variant filtering and annotation) was performed using the OmnomicsNGS analysis software (Euformatics, Finland). Custom variant filter was set up including only non-synonymous variants with coding consequences, read depth greater than 50; benign variants according to the ClinVar database were also excluded [13]. The remaining subset of variants was checked visually, and suspected artefactual variants were excluded.

#### Detection of RB1 deletion by FISH

For the detection of *RB1* loss, the probe ZytoLight® SPEC RB1/13q12 Dual Color Probe (ZytoVision GmbH, Bremerhaven, Germany) was used. The fluorescence in situ hybridization (FISH) procedure was performed as described previously [14].

#### FISH interpretation

One hundred randomly selected nonoverlapping tumor cell nuclei were evaluated in all analyzed samples. *RB1* gene loss was recorded as the number of cells with loss divided by the total number of cells counted. The test was interpreted as positive if >45% of the counted nuclei had gene loss (mean + 3 standard deviation in normal non-neoplastic control tissues).

## Results

### Case presentation

**Fig. 1:**
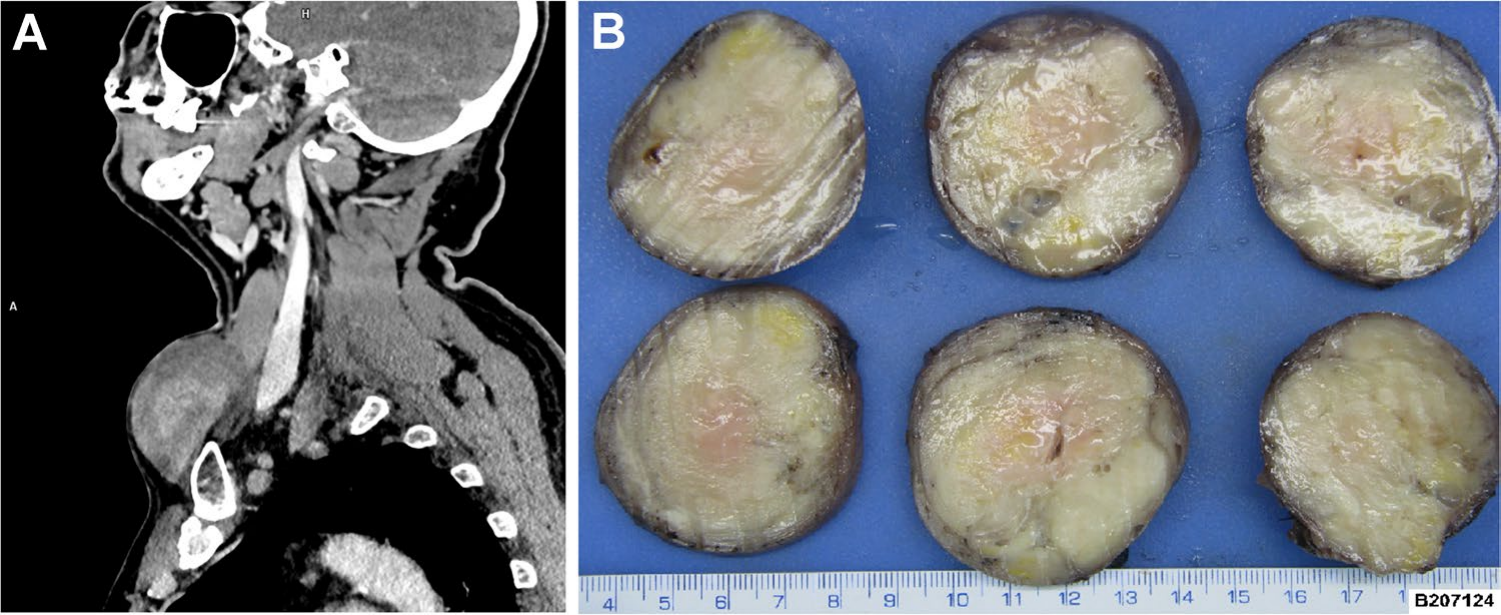
Gross and imaging features. Computed tomography showed a lower neck homogenous mass within the left supraclavicular area near the upper margin of the clavicle which was in close proximity to the tumor (**A**). Grossly, the tumor was oval, well-circumscribed, surrounded by a thin capsule measuring 6 cm in the largest diameter. Cut sections revealed a white to yellowish solid homogenous mass, with fibrillary arrangement containing small cystic-like areas (**B**)

A 78-year-old man without a previous medical history of malignancy presented with a mass in the left supraclavicular area which was surgically excised. The case was sent as a consultation to our department with a diagnosis of metastatic neuroendocrine tumor of unknown origin. Computed tomography of the neck showed a homogenous mass within the left supraclavicular area near the upper margin of the clavicle (Fig. 1A). Grossly, the tumor presented as an oval, well-circumscribed nodule surrounded by a thin capsule measuring 6 × 5 × 5 cm. Cut sections revealed a solid homogenous mass, white to yellowish in color with small cystic areas, but no hemorrhage or necrosis (Fig. 1B). The patient is alive without evidence of recurrence or metastasis 8 months post-surgery.

**Fig. 2:**
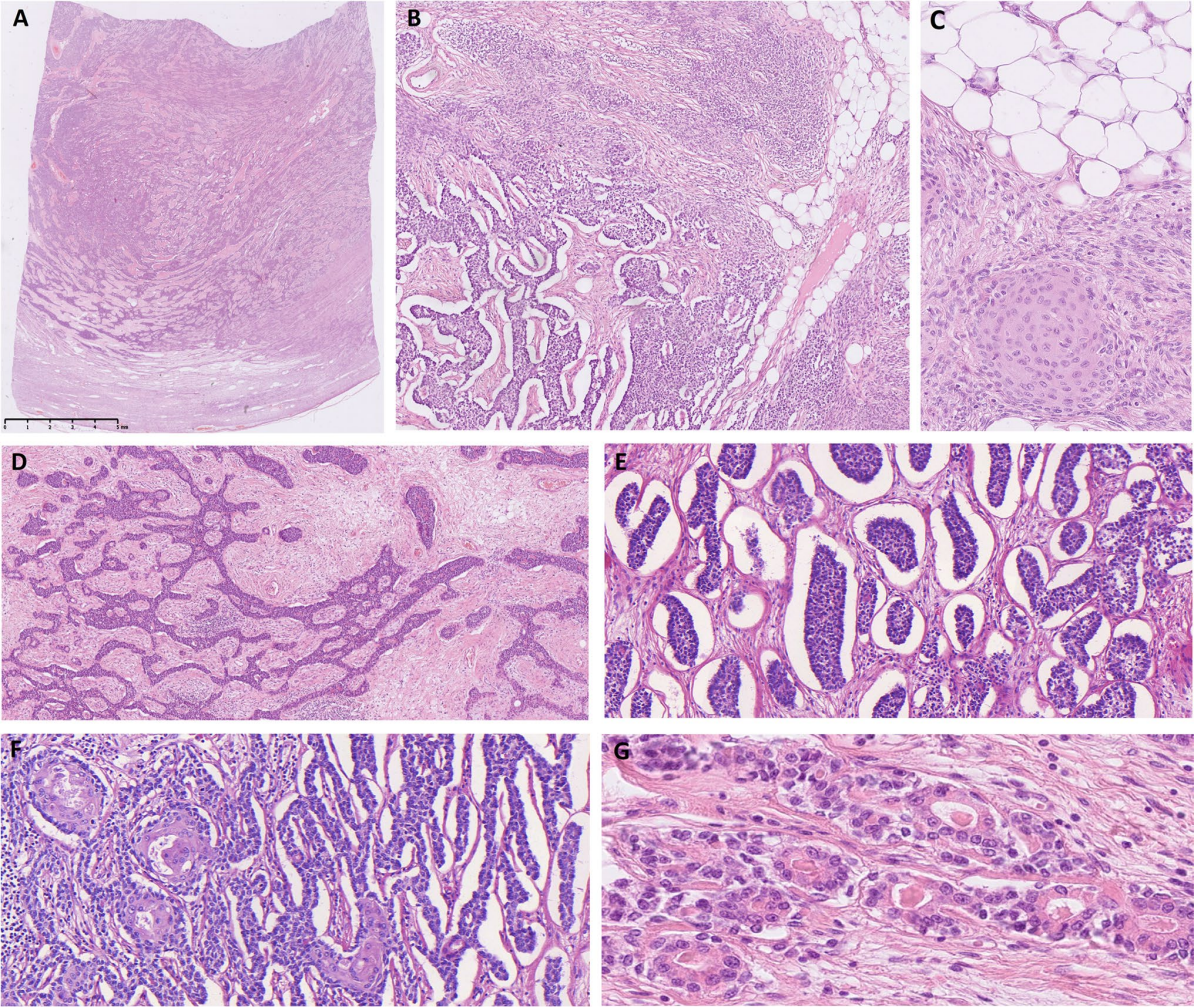
Histological features. The tumor was well-circumscribed. At the periphery, it was composed predominantly of spindle cells (**A**–**C**), while epithelial component reminiscent of neuroendocrine tumor metastasis was present at the central zone (**A**, **B**, **D**–**G**). Fatty tissue was randomly admixed between both components (**B**, **C**). The spindle cells were arranged in haphazard or vague fascicular fashion (**A**, **B**) and had oval nuclei with plump lightly eosinophilic cytoplasm. In some areas, the cells formed solid nests with interspersed fatty tissue (**C**). The epithelial component was either cystic or solid/glandular, the latter showed neuroendocrine-like morphology (**D**–**G**). These areas were arranged in trabecular (**D**) or nested patterns with retraction artifact at the periphery of tumor nests (**E**, **F**). Occasionally, abrupt squamous differentiation was present (**F**). Anastomosing pattern with microglandular formations containing luminal eosinophilic material (**G**)

Histologically, the tumor was well-circumscribed (Fig. 2A), and displayed two components: the first component, which corresponded to classical triphasic pattern of branchioma, showed spindle cells (20% of the tumor), and adipose tissue (5%) entrapping scattered squamoid and cystic epithelial aggregates. This classical component merged with a predominant cellular epithelial component with nested/organoid (neuroendocrine tumor-like) features comprising 75% of the whole mass (Fig. 2B). The spindle cell proliferation was arranged in a storiform, vague fascicular, or haphazard architecture and was localized rather at the periphery of the lesion (Fig. 2A, B). The cells had oval nuclei with light eosinophilic plump cytoplasm, and in some areas, they formed solid plump nests with interspersed fatty tissue (Fig. 2C). Tumor cells were admixed with scant lymphocytes which focally formed lymphoid follicles (not shown). The epithelial component grew either in a cystic formation layered by flattened bilayered epithelial cells (less than 5%) or solid/neuroendocrine tumor-like (more than 95%) architecture (Fig. 2D). The solid areas were composed of middle-sized monomorphic epithelioid cells with regular round to oval nuclei with “salt and pepper” chromatin, distinct nucleoli, and scant pink cytoplasm. These nested/organoid (neuroendocrine-like) areas showed sheets, nests, trabeculae, pseudorosettes/pseudoglandular or microglandular, and interanastomosing patterns (Fig. 2D–G). The anastomosing structures were surrounded by artificially created clefts from the surrounding loosely cellular and mildly vascularized stroma (Fig. 2E, F). Some parts of the tumor were more haphazard with small ducts composed of monomorphic cells with pink cytoplasm, rounded nuclei, and luminal formations sometimes containing dense eosinophilic homogenous material (Fig. 2G). The last component was adipose tissue which was haphazardly dispersed and admixed with the other two components (Fig. 2B, C).

**Fig. 3:**
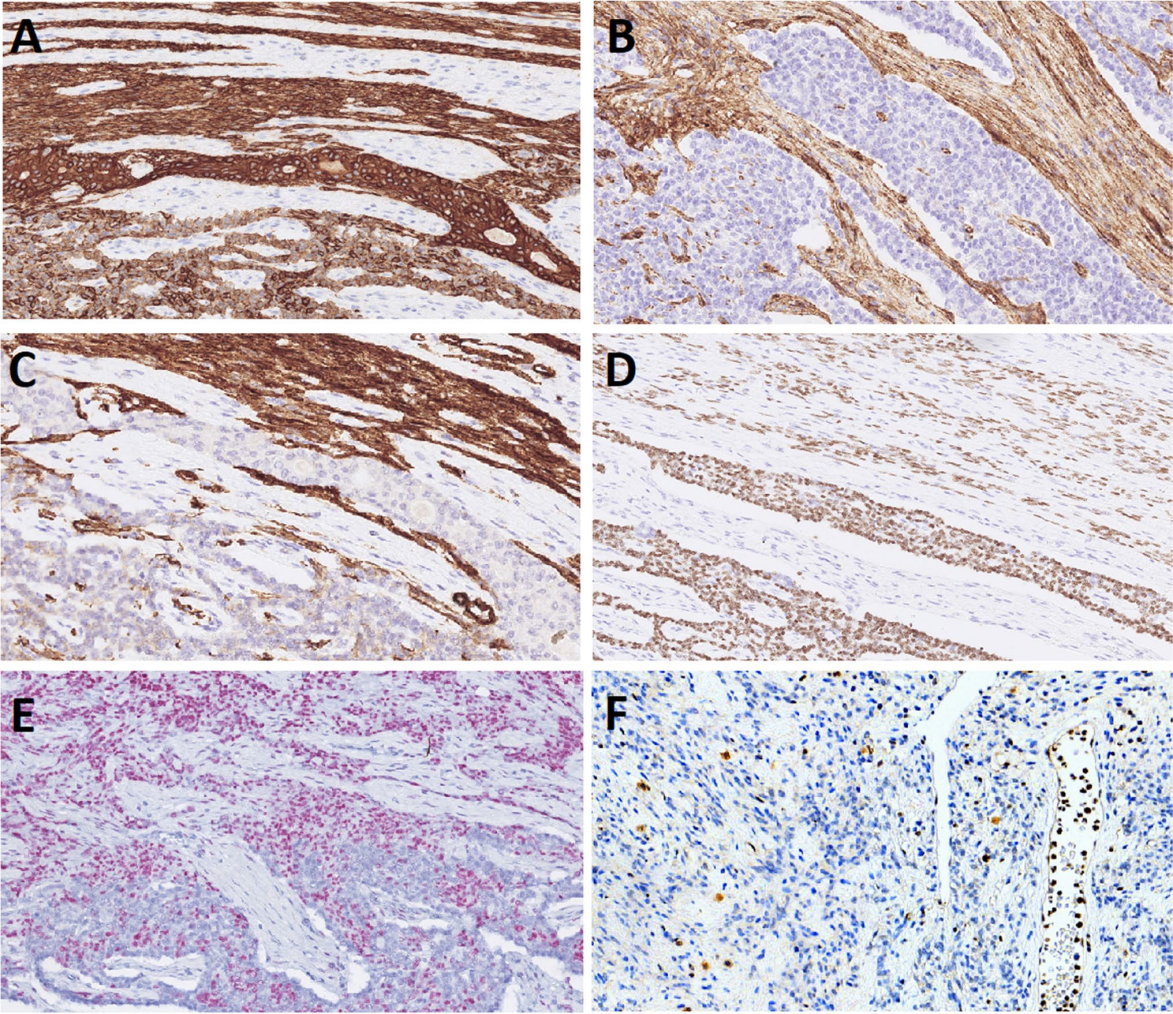
Immunohistochemical features of branchioma. AE1/3 was strongly positive in both the spindle cell and epithelial components (**A**), while CD34 (**B**) and SMA (**C**) were positive in the spindle cells only. p63 showed moderate positivity in spindle cells and was strongly positive the epithelial component (**D**). Androgen receptors were positive in 40% of tumor cells, mainly in the spindle component (**E**). Absent RB1 expression in spindle component with positive internal control in lymphocytes and endothelial cells (**F**)

Immunohistochemically, both spindled and epithelial components were strongly positive for AE1-3 and OSCAR (Fig. 3A). CD34 and SMA were biphasic and highlighted the spindle cell component (Fig. 3B, C). The p63 was expressed focally in spindle cells and stained the epithelial component (Fig. 3D). The nested/organoid (neuroendocrine-like) morphology mirrored the epithelial component immunophenotype. S100 protein was positive in fatty tissue only (not shown). The androgen receptor (AR) was expressed in 40% of tumor cells, predominantly in spindle and solid epithelial components (Fig. 3E). Neuroendocrine markers synaptophysin, chromogranin, INSM1, and CD56 were negative. MSH6 (stained due to the detected molecular alteration, see molecular findings below) was retained in tumor cells. Proliferative activity was low (the MIB1 index in hot-spots reached up to 5%). All components (epithelial, spindle cells, and adipocytes) showed loss of RB1 expression with positive internal control in lymphatic cells and/or endothelium Fig. 3F.

The tumor was tested for *RB1* gene aneuploidy by FISH and underwent molecular genetic testing by TrueSight Illumina Oncology 500 NGS panel. Five pathogenic/likely pathogenic mutations were detected by Illumina TS500, including *MSH6* c.3261dup p.(Phe1088LeufsTer5), *MSH6* c.3202C>T p.(Arg1068Ter), *PTEN* c.385G>A p.(Gly129Arg), *PTEN* c.697C>T p.(Arg233Ter), and *KRAS* c.437C>T p.(Ala146Val), while no aberration of *RB1* gene was found. From one hundred randomly selected nonoverlapping nuclei, 32 nuclei showed monoallelic *RB1* gene loss which was below the cut-off of >45% of the counted cells defined for *RB1* deletion in this study.

## Discussion

Branchioma is a rare lower neck tumor with 85 reported cases to date. The origin of branchioma is controversial; postulated origin from ectopic thymic remnants in the lower neck justified the original terminology “ectopic hamartomatous thymoma” [3]. However, recent investigations point rather toward the branchial cleft apparatus as the most probable origin [4, 5]. Branchioma is not a hamartoma but a true neoplasm most likely derived from the rudimental embryological structures of endoderm and mesoderm, which are responsible for its triphasic morphology including epithelial cells, spindle cells, and adipose tissue [4]. The proportion of each of these components varies from case to case. The spindle cells have haphazard to fascicular arrangement, the epithelial cells grow in cystic, solid, or pseudoglandular patterns, and the adipose component is dispersed throughout the tumor. Both spindle and epithelial cells are immunoreactive with high-molecular-weight cytokeratins, myoepithelial markers (p63 and p40), and the androgen receptor [9]. The spindle cells show reactivity with CD34 and SMA [6, 15] and partially resemble the stroma of spindle cell lipoma [14]. Similar to the latter, immunohistochemical RB1 loss was observed in our case, but no *RB1* genetic alteration was detected. This suggests point mutations and/or other alternate molecular or epigenetic mechanisms responsible for RB1 loss that are not detectable by the FISH method.

Molecular findings in branchioma have been reported in only two studies [8, 10]. One study has looked for *PLAG1* rearrangements to assess distinctness of branchioma from pleomorphic adenoma; no rearrangements have been detected in the 4 analyzed branchiomas [8]. Another study investigated 3 branchiomas using a custom, targeted NGS panel including 1385 pan-cancer-related genes. A hotspot *HRAS* (pGln61Lys) mutation was found in one case of branchioma with intraductal type carcinoma, whereas no definitive oncogenic drivers or copy number alterations were found in the other two cases [10]. We herein expand on the spectrum of molecular findings in branchioma, in which we detected 5 pathogenic/likely pathogenic gene mutations, particularly two *MSH6* mutations, two *PTEN* mutations, and one *KRAS* alteration, indicating molecular heterogeneity in branchioma. Admittedly, we did not perform microsdissection and separate molecular testing of the different tumor components. However, lack of atypia and proliferative features in the nested/organoid (neuroendocrine-like) component suggests it probably represents a morphological variant of the epithelial component. The presence of the retained MSH6 expression in the context of its molecular genetic alteration is not surprising. Loss of MMR proteins precludes heterodimerization of MLH1-PMS2 and MSH2-MSH6. The loss of MSH6 immunoexpression is related to gene mutation with loss of the epitope for the MSH6 antibody. In our case, there was an altered *MSH6* gene but the antibody epitope for MSH6 defective protein was probably retained which resulted in positive nuclear immunoexpression.

Moreover, our case adds to the morphological (prominent neuroendocrine-like nested/organoid features) and immunophenotypic (CD34 expression combined with RB1 loss) heterogeneity/pitfalls related to the differential diagnosis of branchioma. The nested/organoid component retained the epithelial immunophenotype and did not express any of neuroendocrine markers, consistent with a morphological variant of the epithelial part of the tumor and not a dedifferentiation or transdifferentiation. Weissferd and Moran described a series of thymomas with histological neuroendocrine-like differentiation and pancytokeratin positivity but none of the neuroendocrine markers was positive [16], a feature analogous to our current case. Differentiation between thymomas and branchiomas is based on morphology and IHC level. Thymomas are positive for cytokeratins and p63 but show admixture of immature T cell lymphocytes according to subtype and are positive for PAX8 in 55% of cases. However, they are negative with CD34 and SMA.

The lateral neck location together with biphasic tumor cell morphology requires a careful diagnostic approach, especially in fine-needle aspiration biopsies or small biopsy samples. The clinical and radiological differential diagnoses of the adult lateral or anterior neck masses must consider processes of developmental, infectious, or neoplastic nature [17], keeping in mind that over 75% of lower neck masses are likely malignant, mostly metastasis of squamous cell carcinoma or lymphoma [17]. The histological and immunohistochemical differential diagnosis also includes primary or secondary tumors of the epithelial, mesenchymal, neuroepithelial, or neuroendocrine tumor origin.

The combination of cervical localization, variable spindle cell histology, CD34 positivity, and loss of RB1 immunoexpression places branchioma, spindle cell lipoma (SCL), and spindle cell–predominant trichodiscoma (SCPT) in the most common differential diagnosis. SCL is usually localized in the subcutis of the nuchal area of elderly men and is characterized by frequent chromosome 13 and/or 16 deletions, CD34 expression, and RB1 immunonegativity [18]. Androgen receptor expression is a feature shared by both branchioma and SCL, possibly explaining the male predominance in both [19]. Spindle cell–predominant trichodiscoma is mainly face-localized hamartoma of the mantle zone of hair follicle (AKA mantleoma) [20]. In a study by Michalová et al., the authors described 6 cases of SCPT with heterozygous deletion of *RB1* gene, while 18 of 19 cases showed loss of RB1 staining in spindle cells; the morphology of SCPT with *RB1* deletion was indistinguishable from tumors without this genetic alteration [21].

The loss of 13q14 especially in the *RB1* gene is also common in other soft tissue tumors (e.g., cellular angiofibroma or myofibroblastoma of the breast), which together with the overlapping morphology and IHC (loss of RB1 and CD34 positivity) supports the hypothesis of a spectrum of genetically related 13q/*RB1* family tumors. Based on our results, branchioma might represent another potential member of this group. However, studies on larger cohort of branchiomas are needed to investigate this possibility.

Finally, the admixture of epithelial elements and spindle cells might suggest biphasic synovia sarcoma (BSS). Indeed, rare branchioma cases in our experience have been initially judged as low-grade BSS. However, the triphasic morphology, the characteristic location of the tumor, and the immunophenotype are distinctive and rule out BSS. The SS18 immunohistochemistry and molecular testing can help to resolve the issue in equivocal cases or in cases with unusual morphology and lack of lipomatous component.

In summary, we reported an unusual case of branchioma with neuroendocrine-like morphology lacking nuclear RB1 expression and harboring several pathogenic mutations. All these findings underline the wider differential diagnosis of this unusual variant of a rare tumor entity. Additional studies of larger cohorts of branchiomas are needed to investigate whether this immunophenotype and molecular background represent a recurrent feature of these tumors.

### Supplementary information

ESM 1 (DOCX 13 kb)

## Declarations

The sample was used in accordance with ethical guidelines. Informed consent was not required for the study.
