# Supplementary material for: Branchioma with a nested/organoid morphology: molecular profiling of a distinctive potentially misleading variant and reappraisal of potential relationship to CD34-positive/Rb1-deficient tumors of the neck
Source: Virchows Arch. 2023 Jul 4;483(4):541–8. doi: 10.1007/s00428-023-03592-9 (PMC10611845; doi:10.1007/s00428-023-03592-9)
Supplement: Supplementary file 1 — (DOCX 13 kb) [file 428_2023_3592_MOESM1_ESM.docx]

**SAR6**

**FusionPlex Sarcoma custom Kit ArcherDx** detects fusion transcripts in: ALK, BCOR, BRAF, C11orf95, CAMTA1, CCNB3, CDK4, CIC, CSF1, DDX3X, EGFR, EPC1, ETV6, EWSR1, FGFR1, FGFR2, FGFR3, FN1, FOS, FOSB, FOXO1, FOXR2, FUS, GFI1, GFI1B, GLI1, GLI2, HMGA2, CHMP2B, JAZF1, KMT2A, MDM2, MDM4, MEAF6, MET, MGEA5, MKL2, MN1, MYB, MYBL1, MYC, NCOA1, NCOA2, NCOA3, NFIB, NOTCH1, NOTCH2, NOTCH3, NR4A3, NTRK1, NTRK2, NTRK3, NUTM1, PAX3, PDGFB, PDGFRA, PHF1, PIP4K2A, PLAG1, PRDM10, PRDM6, PRKCA, PRKCB, PTEN, RAD51B, RAF1, RB1, RELA, RET, ROS1, RPS19, SERPINE1, SMARCA4, SMARCB1, SRF, SS18, STAT6, TAF15, TCF12, TFE3, TFG, TSC2, TTYH1, USP6, VGLL2, VGLL3, YAP1, YWHAE

and mutations in hotspot genes: ALK, BRAF, CTNNB1, EGFR, FGFR1, FGFR2, FGFR3, MYBL1, MYOD1, NUTM1, PDGFRA, PTEN, RET, ROS1.
